# Repression of tumor necrosis factor-related apoptosis-inducing ligand (TRAIL) but not its receptors during oral cancer progression

**DOI:** 10.1186/1471-2407-7-108

**Published:** 2007-06-25

**Authors:** Nadarajah Vigneswaran, Darryl C Baucum, Jean Wu, Yahuan Lou, Jerry Bouquot, Susan Muller, Wolfgang Zacharias

**Affiliations:** 1 Department of Diagnostic Sciences, The University of Texas Health Science Center at Houston, Dental Branch, Houston, Texas 77030, USA; 2 Departments of Pathology and Otolaryngology–Head and Neck Surgery, Emory University School of Medicine, Atlanta, Georgia 30322, USA; 3 Departments of Medicine, Pharmacology & Toxicology, James Graham Brown Cancer Center, University of Louisville, Louisville, Kentucky 40202, USA

## Abstract

**Background:**

TRAIL plays an important role in host immunosurveillance against tumor progression, as it induces apoptosis of tumor cells but not normal cells, and thus has great therapeutic potential for cancer treatment. TRAIL binds to two cell-death-inducing (DR4 and DR5) and two decoy (DcR1, and DcR2) receptors. Here, we compare the expression levels of TRAIL and its receptors in normal oral mucosa (NOM), oral premalignancies (OPM), and primary and metastatic oral squamous cell carcinomas (OSCC) in order to characterize the changes in their expression patterns during OSCC initiation and progression.

**Methods:**

DNA microarray, immunoblotting and immunohistochemical analyses were used to examine the expression levels of TRAIL and its receptors in oral epithelial cell lines and in archival tissues of NOM, OPM, primary and metastatic OSCC. Apoptotic rates of tumor cells and tumor-infiltrating lymphocytes (TIL) in OSCC specimens were determined by cleaved caspase 3 immunohistochemistry.

**Results:**

Normal oral epithelia constitutively expressed TRAIL, but expression was progressively lost in OPM and OSCC. Reduction in DcR2 expression levels was noted frequently in OPM and OSCC compared to respective patient-matched uninvolved oral mucosa. OSCC frequently expressed DR4, DR5 and DcR1 but less frequently DcR2. Expression levels of DR4, DR5 and DcR1 receptors were not significantly altered in OPM, primary OSCC and metastatic OSCC compared to patient-matched normal oral mucosa. Expression of proapoptotic TRAIL-receptors DR4 and DR5 in OSCC seemed to depend, at least in part, on whether or not these receptors were expressed in their parental oral epithelia. High DR5 expression in primary OSCC correlated significantly with larger tumor size. There was no significant association between TRAIL-R expression and OSSC histology grade, nodal status or apoptosis rates of tumor cells and TIL.

**Conclusion:**

Loss of TRAIL expression is an early event during oral carcinogenesis and may be involved in dysregulation of apoptosis and contribute to the molecular carcinogenesis of OSCC. Differential expressions of TRAIL receptors in OSCC do not appear to play a crucial role in their apoptotic rate or metastatic progression.

## Background

TRAIL (tumor necrosis factor-related apoptosis-inducing ligand; Apo2L) is a type II transmembrane protein which selectively induces apoptosis in tumor cells but not normal cells [1,2]. Because of this differential sensitivity TRAIL is considered an ideal anticancer drug [3,4]. It interacts with four distinct surface receptors, TRAIL-R1 (DR4), -R2 (DR5), -R3 (DcR1), and R4 (DcR2) and with the soluble receptor osteoprotegerin [1,2]. DR4 and DR5 act as transmembrane signaling death receptors with cytoplasmic death domains (DD) which respond to ligand binding and activate the extrinsic cell death pathway by facilitating interaction between the specific adapter protein (FAS-associated DD) and proapoptotic effector proteins (caspases 8 and 10) [5]. DcR1 and DcR2 (decoy receptors), conversely, cannot mediate apoptosis because they lack functional DD [6]. It has been suggested that the differences in the expression levels of death (DR4 and DR5) versus decoy (DcR1 and DcR2) receptors can determine the sensitivity of tumor cells to TRAIL-induced apoptosis [6]. However, susceptibility to TRAIL-induced apoptosis does not always correlate well with the cell surface expression levels of death-inducing and decoy TRAIL receptors suggesting a regulatory role for downstream signaling and effector molecules [7,8]. Furthermore, recent studies indicate that TRAIL susceptibility of tumors *in vivo *is modified by tumor microenvironmental factors and tumor hypoxia [9,10].

Recombinant TRAIL protein (rTRAIL) induces significant tumor regression in mice bearing human tumor xenografts without producing any serious systemic effects in the host [11,12]. Importantly, radiation and certain chemotherapeutic drugs increase the susceptibility of tumor cells to rTRAIL and agonistic TRAIL-R antibodies [13-17]. Hence, combinations of rTRAIL + chemotherapy or TRAIL + radiation produce synergistic anti-tumor effects both *in vitro *and in mice bearing human tumor xenografts [18]. Human rTRAIL (PRO1762) and monoclonal antibodies that induce trimerization of DR4 (HGS-ETR 1) and DR5 (HGS-ETR 2) are currently undergoing phase Ib and II clinical evaluations, respectively [19]. Moreover, use of DR4 (HGS-ETR1) agonist antibody as a single agent or in combination with chemotherapy has been shown to stabilize disease in patients with advanced head and neck cancer [20]. In addition to its therapeutic potential, endogenously expressed TRAIL is an effector molecule important for the host's antitumor immune response [2,21,22].

Despite various treatment approaches, most patients with advanced oral squamous cell carcinomas (OSCC) develop local or regional recurrences (50–60%) and metastatic disease (~20%) leading to poor survival rate. Therefore, there is an unmet need for more efficacious and less toxic molecularly targeted therapies for treating OSCC. Recent preclinical and clinical data have shown the potential utility of TRAIL-R targeted therapies in advanced cancers, including OSCC [4,19,23]. Currently, there are only limited data available pertaining to the baseline expression levels of TRAIL and its receptors in normal oral mucosa, oral premalignancies (OPM) and primary or metastatic oral squamous cell carcinoma (OSCC) [24,25]. These data are critical to analysis and interpretation of clinical trial data involving rTRAIL and TRAIL receptor agonist antibodies in OSCC patients, as well as the understanding of the role of TRAIL and its receptors during oral carcinogenesis. In the present study we determined changes in the expression of TRAIL and TRAIL-R during OSCC development and progression by comparing expression patterns in normal oral mucosa (NOM), oral premalignancies (OPM), primary OSCC and metastatic OSCC. We also correlated the expression levels of TRAIL-receptor in OSCC with various clinicopathologic prognosticators. Finally we correlated the apoptosis rate of tumor cells and tumor-infiltrating lymphocytes (TIL) in OSCC with their respective TRAIL and TRAIL-R expression patterns.

## Methods

### Cell lines and human tissue

Normal human oral mucosal epithelia cells (NOM) were established from discarded human gingival tissue. NOM cells within their first two serial passages in culture were used for RNA isolation and cellular protein extraction. Details related to OPM (Leuk1 & Leuk2), primary OSCC (686Tu, 1386Tu and 101A) and metastatic OSCC (686Ln and 1386Ln) cell lines have been described previously [7,26-28]. HOK-16B is a normal oral keratinocyte-derived cell line immortalized by transfection with HPV-16 genome [29]. Chronic exposure of HOK-16B cells with benzo (*a*) pyrene produced the tumorigenic HOK-16B-Bap-T cell line [29]. NOM, Leuk1, Leuk2 and HOK-16B cells were grown in keratinocyte growth media (KGM-2) supplemented with growth factor Bullit kit (Cambrex, East Rutherford, NJ, USA). HOK-16B-BaP-T and OSCC cell lines were maintained in DMEM/F12 50/50 mix (Cambrex) containing 10% fetal bovine serum, 0.4 μg/ml hydrocortisone, and penicillin-streptomycin-amphotericin antibiotic mix.

Archival tissue specimens from primary OSCC (n = 45) and corresponding lymph node metastases (n = 11) were used for light microscopic and immunohistochemical studies (Table 1). Oral leukoplakias with moderate to severe dysplasia (n = 25) were used as OPM specimens. Five μm serial sections were cut from each specimen and processed for H&E and immunohistochemical staining. The histological grading of OPM and OSCC was determined by two pathologists using H&E stained sections according to published criteria [30]. All tissue specimens and appropriate clinical information were obtained under the guidelines and approval of the Institutional Review Boards of The University of Texas Health Science Center at Houston.

**Table 1:** Clinical data of the OSCC specimens used in this study

Parameter	No of cases (%)
**Median age**: 42 Y	42 (100)
**Age range **(years): 37–81 Y	
**Sex**	
Male	29 (69)
Female	13 (31)

**Site**	
Buccal mucosa	2 (5)
Tonsillar area	7 (17)
Retromolar pad	3 (7)
Gingiva	2 (5)
Floor of the mouth	8 (19)
Tongue	15 (36)
Unknown	5 (11)

### Microarray data sets

We interrogated the expression levels of TRAIL and its receptors in our pre-existing microarray data sets on the above oral cell lines representing different phenotypes. Relative RNA expression levels were determined using Affymetrix U133A oligonucleotide microarrays (33,000 annotated genes) as described [27,28]. Either two or four biological replicate experiments were done independently by two different investigators. The U133A array included three different probe sets for TRAIL (NM_003810), DR5 (TRAIL-R2; NM_003842) and DcR1 (TRAIL-R3; NM_003841) and one probe set for DcR2 (TRAIL-R4; NM_003840), but no probe sets for TRAIL receptor DR4 (TRAIL-R1; NM_003844). Signal intensity for each gene was calculated using the Affymetrix MAS 5.0 probe level algorithm. Phenotypic-specific gene expression values for TRAIL, DR5, DcR1 and DcR2 genes were calculated by averaging signal intensities across sample replicates for cell lines belonging to each phenotypic group.

### Western blotting for TRAIL protein

TRAIL protein levels were examined by Western blotting in cell extracts of NOM cells from two different patients (NOM-1 & 2), OPM cell line Leuk1, and from primary (1386Tu) and metastatic (1386Ln) OSCC cell lines. These cellular extracts were separated by 12% SDS-PAGE and transferred to nitrocellulose membrane (Novex, San Diego, CA). The membranes were incubated in blocking solution (5% milk powder in 10 mM TRIS-HCl, pH 8.0, 150 mM NaCl, 0.1% Tween 20) at room temperature for 1 h, then immunoblotted with polyclonal goat anti-human TRAIL (1: 200, Santa Cruz Biotechnology, CA, USA) or monoclonal mouse anti-βactin (Sigma-Aldrich Co, St. Louis, MO) antibody followed by horseradish peroxidase-conjugated secondary antibody and chemiluminescence detection.

### Immunohistochemistry

We investigated TRAIL and its receptor expression by semiquantitative immunohistochemistry in 25 OPMs (leukoplakias with moderate to severe dysplasia) and in 45 primary OSCCs and 9 patient-matched metastatic OSCCs. Tissue sections were deparaffinized, rehydrated, and subjected to antigen retrieval by heating in target antigen retrieval solution (DakoCytomation, Carpinteria, CA, USA) according to the manufacturer's protocol. Polyclonal antibodies against human TRAIL (goat anti-TRAIL, Santa Cruz, CA. USA; 1:40), DR4 (goat anti-DR4, Santa Cruz, CA., USA; 1:50), DR5 (rabbit anti-DR5, Oncogene, CA., USA; 1:100), DcR1 (rabbit anti-DcR1, Oncogene, CA, USA; 1:50) and DcR2 (rabbit anti-DcR1, Oncogene, CA, USA; 1:25) were used for immunohistochemical detection. Apoptotic rates of OSCC cells and tumor-infiltrating lymphocytes (TIL) were determined by immunohistochemical detection of cleaved caspase 3 (Rabbit anti-cleaved caspase 3, Cell Signaling, CA, USA, 1:50) [31]. Immunoreactive sites were visualized using the standard Streptavidin-Biotin-HRP detection method, using diaminobenzidine tetrachloride as chromogenic substrate.

### Pathological and immunohistochemical evaluations

Immunoreactive patterns of TRAIL and its receptors were compared with uninvolved mucosa adjacent to the epithelial dysplasias and invasive tumors. Expression levels of TRAIL and its receptors were evaluated semiquantitatively by two independent examiners as described [32]. A score from 0 (no staining) to 4 (strong immunoreactivity) was assigned to staining intensity, and the percentage of positive cells for each staining intensity in tumors and dysplastic areas of leukoplakias was determined. Immunoreactive scores (range 0 – 4) were calculated by multiplying percentage of positive cells times staining intensity score. Cleaved caspase-3 positive tumor cells and TIL in randomly selected five to seven high-power (200 ×) fields (HPF) across the tumor section were counted using Image Pro Plus V (Media Cybernetics, Silver Spring, MD, USA). Caspase 3 labeling index (LI_Casp_) for each tumor is expressed as average # positive cells/HPF.

### Statistical analysis

Two-sample t-test and one-way ANOVA were used to determine the statistical significance of the differences in TRAIL and its receptors expression levels in OPM and OSCC with various clinicopathologic parameters. Pearson's correlation was used to test the relationship between TRAIL-R expression patterns of uninvolved oral mucosa, and primary and metastatic tumor from the same patient.

## Results

### TRAIL, DR5, DcR1 and DcR2 RNA expression levels in normal, premalignant and malignant oral epithelial cells *in vitro*

**Figure 1:**
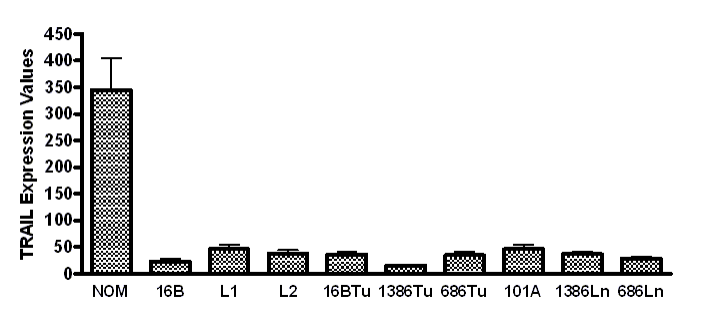
TRAIL mRNA levels are markedly higher in normal oral epithelial cells (NOM) in comparison to immortalized (16B), premalignant (L1 and L2) and malignant oral epithelial cells (16BTu, 1386Tu, 686Tu, 101A, 1386Ln and 686Ln). Relative RNA expression levels of TRAIL were determined using Affymetrix U133A oligonucleotide microarrays. Signal intensity for TRAIL was calculated using the Affymetrix MAS 5.0 probe level algorithm. **NOM**: Normal oral epithelial cells; **16B**: Normal oral epithelial cells immortalized by HPV-16 transfection (HOK-16B). **L1**: OPM cell line MSK-Leuk1; **L2**: OPM cell line MSK-Leuk2; **16BTu**: tumorigenic cell line derived from HOK-16B; (HOK-16B-Bap-T); **1386Tu, 686Tu and 101A**: Cell lines derived from primary OSCC. **1386Ln and 686Ln**: Cell lines derived from synchronous lymph node metastases of 1386Tu and 686Tu tumors.

**Figure 2:**
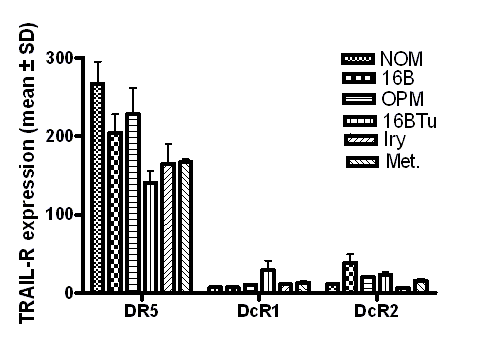
Expression levels of TRAIL-R, DR5, DcR1 and DcR2 are not significantly different among normal (NOM), immortalized (16B), premalignant (OPM), and malignant primary (Iry) and metastatic (Met) oral epithelial cells. Relative RNA expression levels of TRAIL-R were determined using Affymetrix U133A oligonucleotide microarrays and their signal intensities were calculated using the Affymetrix MAS 5.0 probe level algorithm. Phenotypic-specific gene expression values for DR5, DcR1 and DcR2 genes were calculated by averaging signal intensities across sample replicates for cell lines belonging to each phenotypic group. The mRNA levels of DR5 are significantly higher than the DcR1 and DcR2 mRNA levels in these cell lines. The DNA microarray chips used in this study did not have the probe sets for DR4. **NOM**: Normal oral epithelial cells; **16B**: Immortalized normal oral epithelial cells; **OPM**: Average target intensities of premalignant cell lines MSK-Leuk1 and MSK-Leuk2; **16BTu**: tumorigenic cell line derived from HOK-16B; **Iry**: Average target intensities of primary OSCC cell lines 1386TU, 686Tu and 101A. **Met**: Average target intensities of metastatic OSCC cell lines 1386Ln and 686Ln.

**Figure 3:**
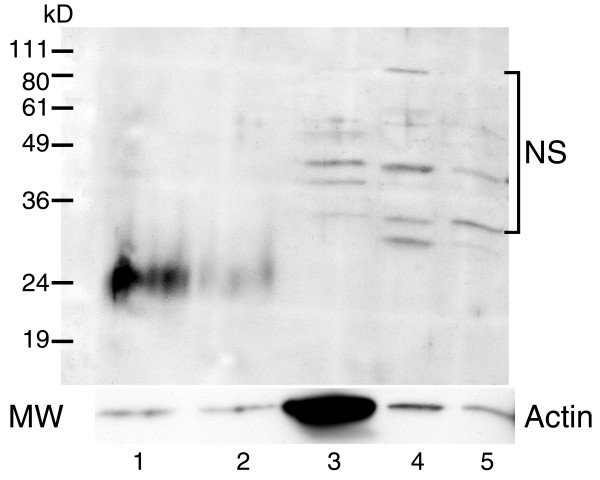
TRAIL protein is detectable only in normal oral epithelial cells (lanes 1 and 2) but not in premalignant (lane 3) and malignant oral epithelial cell lines (lanes 4 and 5). Total cellular proteins were extracted from two normal oral epithelial samples (NOM-1 and 2-lanes 1 and 2), OPM cell line (MSK-Leuk1-lane 3), primary OSCC cell line (1386Tu-lane 4) and metastatic OSCC cell line (1386Ln-lane 5) and analyzed for TRAIL protein by Western blotting. **NS**: none specific bands.

TRAIL mRNA was detectable in all samples; however its expression level was significantly (p ≤ 0.001) higher in NOM cells (mean ± SD = 364.9 ± 20.4) compared to immortalized oral epithelia (HOK-16B; mean ± SD = 23.3 ± 10.6), OPM (Leuk1 and Leuk 2 cells mean ± SD = 48.2 ± 20.1), malignant primary (686Tu, 1386Tu, HOK-16B-Bap-Tu &101A; mean ± SD = 25.3 ± 11.3) and metastatic (686Ln and 1386Ln; mean ± SD = 32.5 ± 4.8) oral epithelial cells (Figure 1). Among the TRAIL receptors, DR5 mRNA was consistently higher (> 5-fold) in all cell lines compared to low mRNA levels of DcR1 and DcR2 (Figure 2). DR4 expression levels were not available in our microarray database. There were no significant differences in the expression levels of all three TRAIL receptors among normal, immortalized, premalignant, and malignant primary and metastatic oral epithelial cells (Figure 2). Western blotting analysis showed that TRAIL protein is detectable as a 24 kD band only in NOM cells but not in OPM and OSCC cells (Figure 3). We previously reported that expression of DR4, DR5, DcR1 and DcR2 proteins in primary and metastatic OSCC cells were not significantly different [7].

### Expression of TRAIL and its receptors in OPM specimens

**Figure 4:**
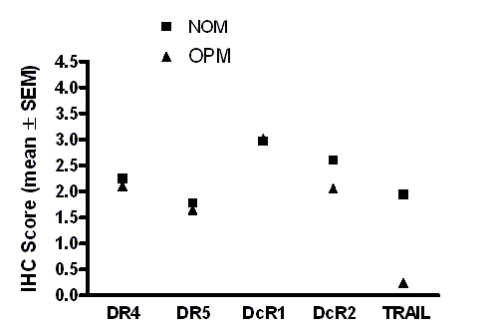
The expression levels of TRAIL and DcR2 are markedly reduced in oral premalignancy compared to normal oral mucosa. Graphic depiction of the mean expression levels of TRAIL and TRAIL-R in patient matched normal oral mucosa (NOM), oral premalignancies (OPM: leukoplakias with dysplasia). Mean expression levels of DR4, DR5 and DcR1 did not differ significantly between normal oral mucosa (NOM) and oral premalignancies (OPM).

**Figure 5:**
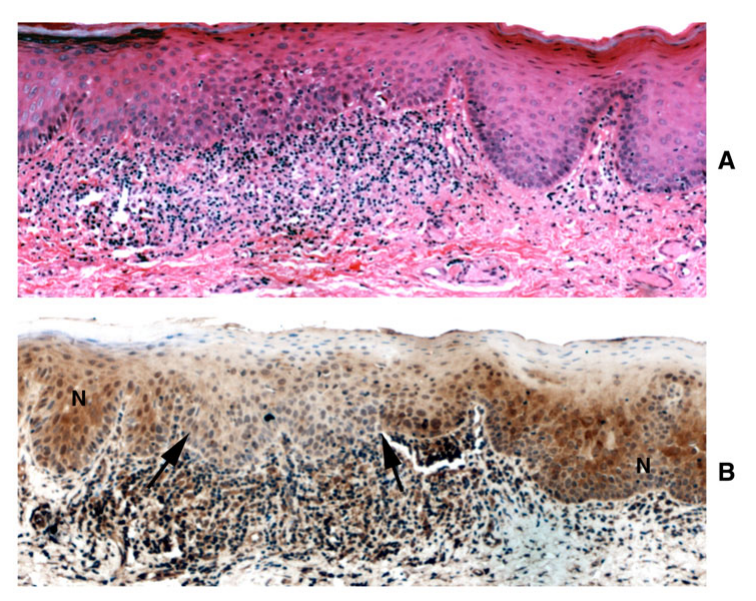
Loss of TRAIL expression in oral leukoplakia with moderate epithelial dysplasia. A: Hematoxylin and eosin staining; B: Immunohistochemical staining for TRAIL (× 100). Note that immunoreactivity for TRAIL is significantly reduced in dysplasia (arrows) compared to adjacent uninvolved mucosa (N).

**Figure 6:**
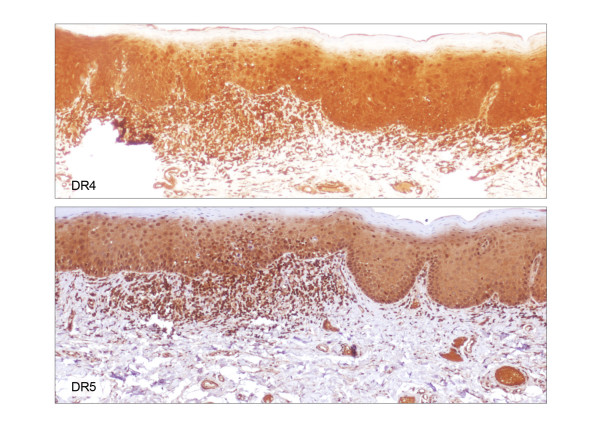
DR4 and DR5 immunohistochemical staining of the lesion shown in figure 5 (× 100). Immunoreactivity for either DR4 (top) or DR5 (bottom) is not altered in dysplasia compared to adjacent uninvolved mucosa.

**Figure 7:**
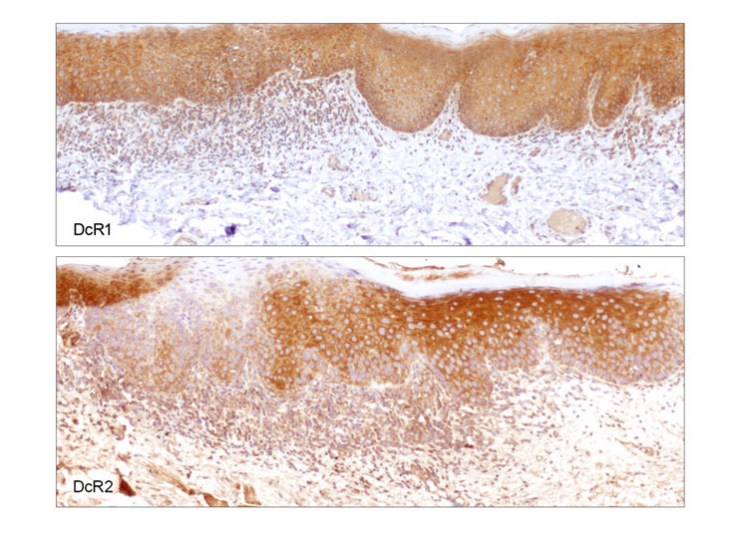
DcR1 and DcR2 immunohistochemical staining of the lesion shown in figure 5 (× 100). Immunoreactivity for DcR1 (top) is not altered in dysplasia compared to adjacent uninvolved mucosa. Focal loss of DcR2 expression (bottom) is noted in dysplasia compared to adjacent uninvolved mucosa.

**Figure 8:**
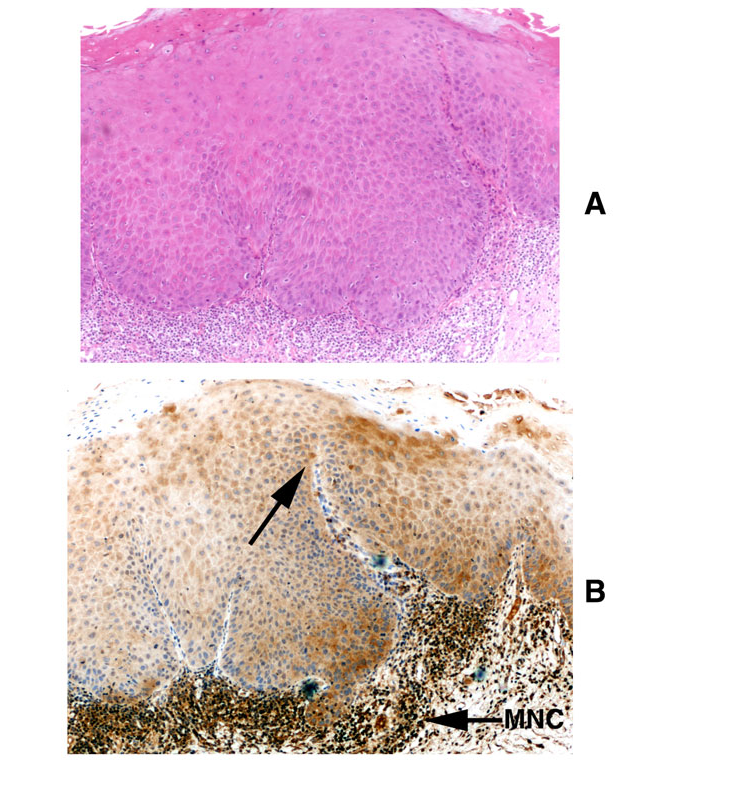
Loss of TRAIL expression pattern in oral leukoplakia with severe dysplasia. A: Hematoxylin and eosin staining (× 100); B: Immunohistochemical staining for TRAIL (× 100). Note that TRAIL expression is mostly lost in dysplastic epithelium (arrow). In contrast, mononuclear immune cell infiltrate (MNC) associated with dysplasia reveals intense staining for TRAIL.

**Figure 9:**
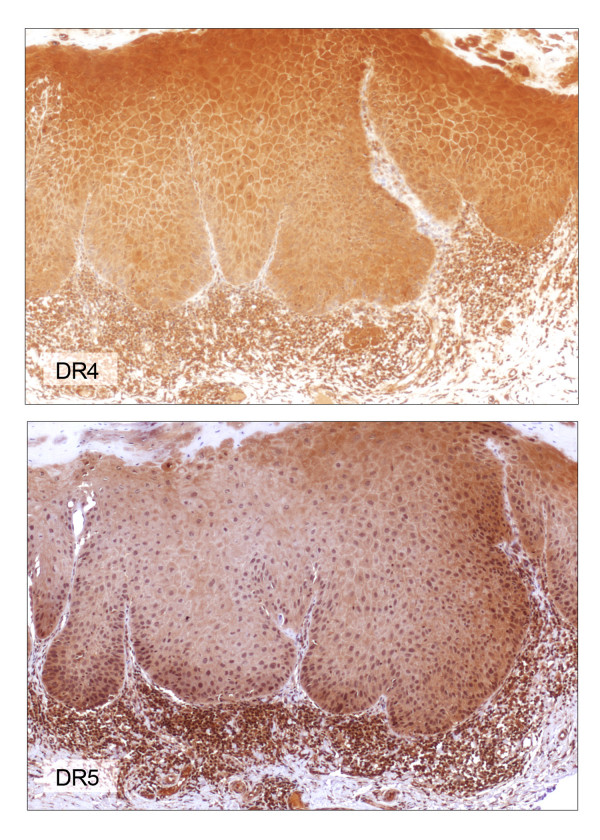
DR4 and DR5 immunohistochemical staining of the lesion shown in figure 8 (× 100). Dysplastic epithelium and associated mononuclear immune cell infiltrate (MNC) demonstrate intense staining for both DR4 and DR5.

**Figure 10:**
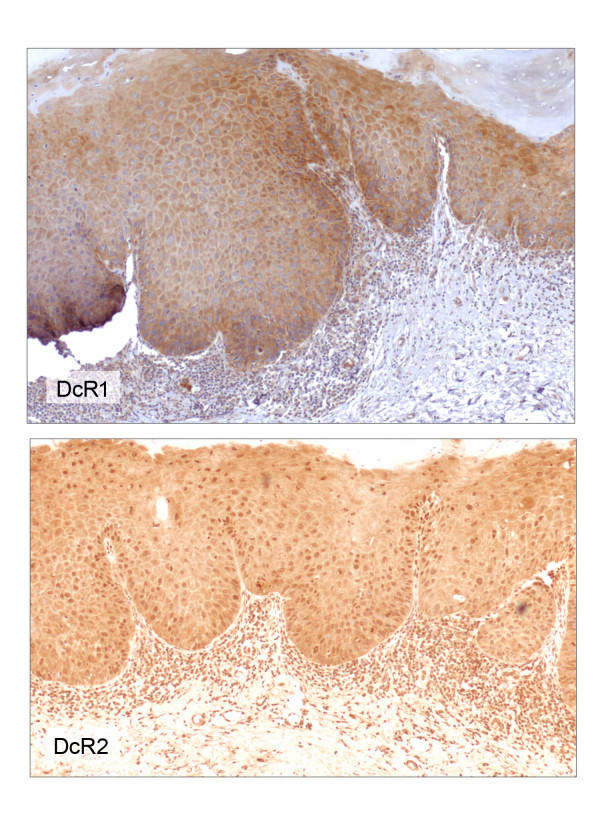
DcR1 and DcR2 immunohistochemical staining of the lesion shown in figure 8 (× 100). Dysplastic epithelium and associated mononuclear immune cell infiltrate (MNC) are positive for both DcR1 and DcR2.

All OPM specimens (leukoplakias/erythroplakias with moderate to severe dysplasia) used in this study had normal oral mucosa (NOM) adjacent to the dysplasia. Of the 25 cases of oral leukoplakias with dysplasia, 56% and 44% of them had moderate and severe dysplasia, respectively. Stratified squamous epithelium of the NOM exhibited membrane and cytoplasmic immunoreactivity for DR4, DR5, DcR1 and DcR2 (Figures 4, 5, 6, 7, 8, 9, 10). Cytoplasmic TRAIL protein was constitutively expressed by NOM (Figures 4, 5). Moreover, DR4, DR5 and DcR1 were expressed in all dysplastic oral epithelia (Figures 4,6, 7). On the other hand, TRAIL expression was lost in dysplasia compared to adjacent NOM (Figures 4, 5, 8). Staining intensity for DcR2 was also lower in dysplasia than in adjacent NOM (Figures 4 &7).

### Expression of TRAIL and TRAIL-R in primary and metastatic OSCC specimens

**Figure 11:**
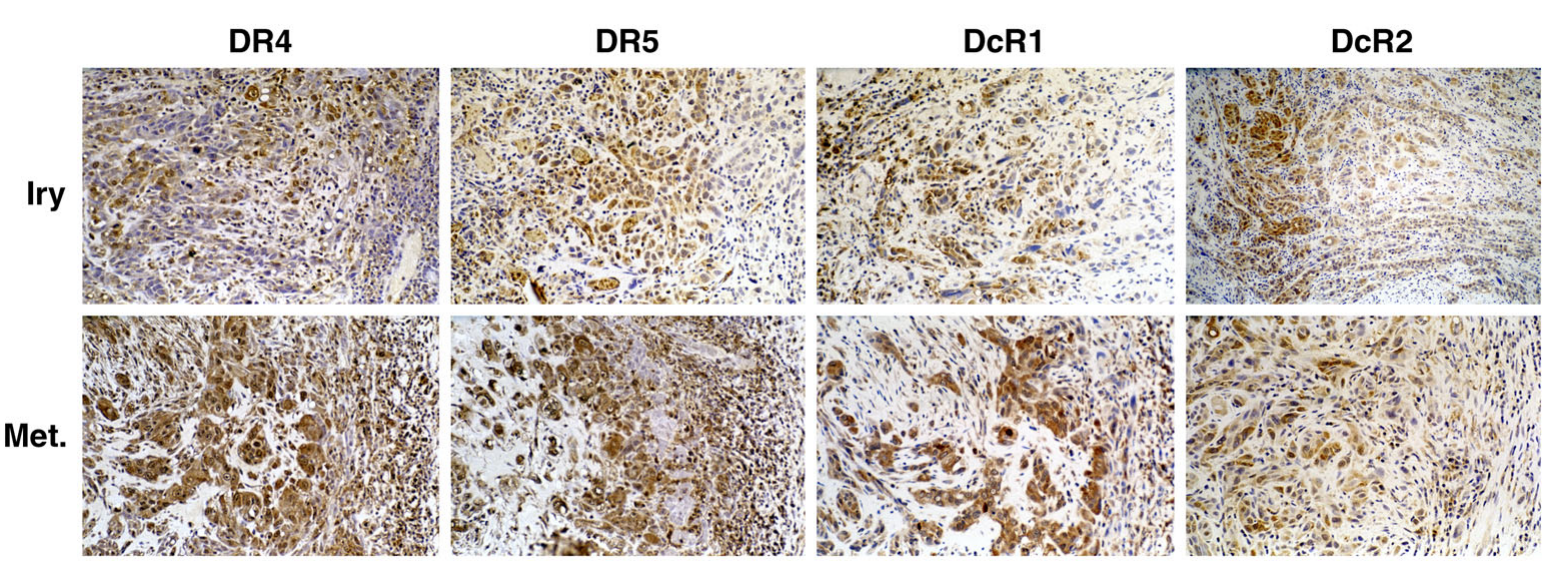
Expression patterns of TRAIL-R, DR4, DR5, DcR1 and DcR2 are not different between primary and synchronous metastatic OSCC. Immunohistochemical expression patterns of TRAIL receptors in primary (Iry) and lymph node metastasis (Met.) of OSCC from the same patient. (× 200).

Forty-two primary OSCC and nine patient-matched cervical lymph node metastases, including NOM adjacent to the primary OSCC, were examined for TRAIL and TRAIL-R expression patterns (Table 1). Expression levels of each TRAIL-R in uninvolved oral mucosa adjacent to primary OSCC were determined and their Pearson correlation coefficient was calculated to be 0.898 for DR4, 0.863 for DR5 and 0.754 for DcR1 with a two-tailed *P *value of < 0.001, indicating a strong correlation in their expression patterns between primary tumors and matched uninvolved oral mucosae. A similar correlation was not observed for DcR2. TRAIL protein was completely negative in 27% of the OSCC cases examined; the remaining OSCC revealed only a few (< 5%) isolated tumor cells with cytoplasmic staining for TRAIL (Data not shown). TRAIL-R in primary OSCC demonstrated marked inter- and intratumoral heterogeneity in their staining patterns. DR4 was expressed in 98% of OSCC (41/42 cases) (IHC score: mean ± SD = 2.24 ± 0.918; range 0.5 – 4) and 57% of these tumors exhibited high levels of DR4 expression (IHC score ≥ 2) (Figure 11). Thirty-nine of 42 OSCC cases (93%) were positive for DR5 (IHC score: mean ± SD = 1.362 ± 0.696; range 0.5 – 3.2) and 21% of these tumors were high expressors (IHC score ≥ 2) for DR5 (Figure 11). All OSCC were positive for either DR4 or DR5 and none of them were negative for both receptors. Among the TRAIL decoy receptors, DcR1 was expressed in all OSCC cases (IHC score: mean ± SD = 2.52 ± 0.674; range 1.0 – 4) and 71% of these cases expressed high levels (IHC score ≥ 2) of DcR1 (Figure 11). On the other hand, DcR2 was positive in 62% (26/42) of OSCC cases (IHC score: mean ± SD = 0.628 ± 0.483; range 0.5 – 2.3) but was expressed in high levels only in one case (Figure 11). Expression levels of DR4, DR5, DcR1 and DcR2 in metastatic OSCC were not significantly different from their patient-matched primary tumors (Figure 11).

### TRAIL-R expression levels and clinicopathologic parameters

We examined the relationship between TRAIL-R expression and various prognostic factors. There was no significant association of either DR4 or DcR1 or DcR2 expression with either tumor size (T) or nodal status (N) or histologic grade of the primary tumor (Table 2). Expression levels of DR5 in primary OSCC correlated positively with tumor size (*p = 0.03*) but failed to show any significant correlation with either nodal status or histologic tumor grade (Table 3).

**Table 2:** Correlation between TRAIL receptor expression levels and clinicopathologic prognosticators in primary OSCC

Variables	DR4 Mean ± SD	DR5 Mean ± SD	DcR1 Mean ± SD	DcR2 Mean ± SD
**Histologic grade**				
Well differentiated (n = 6)	2.96 ± 0.53	1.23 ± 0.71	2.40 ± 1.05	0.55 ± 0.38
Mod. differentiated (n = 19)	2.03 ± 0. 91	1.51 ± 0.79	2.46 ± 0.65	0.69 ± 0.42
Poorly differentiated (n = 8)	2.15 ± 0.86	1.01 ± 0.26	2.61 ± 0.61	0.80 ± 0.73
Basaloid SCC (n = 9)	2.18 ± 1.05	1.43 ± 0.70	2.64 ± 0.54	0.39 ± 0.36
*P *value	*0.189*	*0.366*	*0.864*	*0.305*

**Tumor size**				
pT1 (n= 14)	2.45 ± 1.06	1.19 ± 0.65	2.41± 0.57	0.53 ± 0.47
pT2 (n = 23)	2.16 ± 0.85	1.44 ± 0.63	2.60 ± 0.77	0.60 ± 0.41
pT3 (n = 2)	2.05 ± 0.21	2.5 ± 0.98	2.45 ± 0.07	0.75 ± 0.07
pT4 (n = 3)	1.93 ± 1.15	2.7 ± 0.57	2.7 ± 0.7	1.10 ± 1.04
*P *value	*0.734*	*0.03*	*0.846*	*0.336*

**Lymph node status**				
pN0 (n = 25)	2.27 ± 0.88	1.36 ± 0.79	2.67 ± 0.75	0.69 ± 0.55
pN1/2 (n = 17)	2.21 ± 0.99	1.35 ± 0.57	2.34 ± 0.50	0.53 ± 0.36
*P *value	*0.842*	*0.986*	*0.098*	*0.256*

**Table 3:** Correlation between TRAIL/TRAIL-R expression patterns and apoptosis rates of tumor cells and tumor-infiltrating lymphocytes within primary oral squamous cell carcinomas

TRAIL/TRAIL-R	LI_CASP_-TU Mean ± SEM	LI_CASP_-LY Mean ± SEM
**TRAIL**		
Focally positive tumors (n = 31; 73%)	3.43 ± 0.5	5.95 ± 0.74
Negative tumors (n = 11; 27%)	3.20 ± 0.83	10.91 ± 2.9
*P *value	*0.818*	*0.145*

**DR4**		
High expressors (IHC score > 2; n = 28; 67%)	2.89 ± 0.47	5.80 ± 0.8
Low expressors (IHC score < 2; n = 14; 33%)	4.4 ± 0. 93	9.24 ± 2.2
*P *value	*0.11*	*0.07*

**DR5**		
High expressors (IHC score > 2; n = 9; 21%)	2.4 ± 0.57	7.4 ± 3.01
Low expressors (IHC score < 2; n = 33; 79%)	3.61 ± 0.51	6.73 ± 0.81
*P *value	*0.24*	*0.75*

**DcR1**		
High expressors (IHC score > 2; n = 36; 86%)	3.55 ± 0.48	6.56 ± 0.77
Low expressors (IHC score < 2; n = 6; 14%)	3.53 ± 1.32	8.2 ± 3.4
*P *value	*0.99*	*0.48*

**DcR2**		
High expressors (IHC score > 2; n = 1; 2%)	12.2	20.2
Low expressors (IHC score < 2; n = 41; 98%)	3.15 ± 0.37	6.5± 0.78
*P *value	*N/A*	*N/A*

**Histology**		
Conventional squamous cell carcinoma (n = 33)	2.67 ± 0.62	2.67 ± 0.62
Basaloid squamous cell carcinoma (n = 9)	3.62 ± 0.51	11.27 ± 2.21
*P *value	*0.36*	*0.013*

### TRAIL-R expression levels and apoptosis rates in tumor cells and tumor infiltrating lymphocytes (TIL)

**Figure 12:**
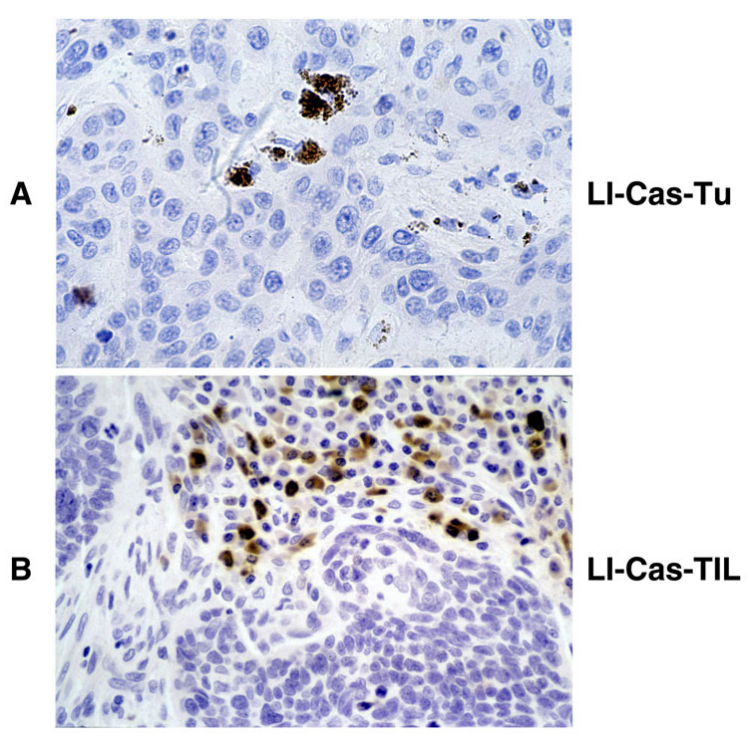
Immunohistochemical staining for cleaved caspase 3 to detect apoptotic cells among tumor cells and tumor-infiltrating lymphocytes. Examples showing the detection apoptotic cells among tumor cells (LI-Cas-Tu)) and TIL (LI-Cas-TIL) based on their immunoreactivity for cleaved caspase-3 (× 400).

We determined the apoptosis rates of tumor cells and TIL by measuring their cleaved caspase 3 labeling indices (LI_Casp_) in primary OSCC (Figure 12). The overall LI_Casp _of tumor cells was 3.4/HPF (LI_Casp_-TU = 3.4 ± 2.6; range = 0.2 – 12.2), and LI_Casp _of TIL was 6.9/HPF (LI_Casp_-TIL = 6.9 ± 5.2; range = 0.6–25). There were no significant associations between the tumor cells or TIL apoptosis rates and tumor size (T) or nodal status (N) (Table 3). However, basaloid squamous cell carcinoma, which is a highly aggressive variant of OSCC, exhibited significantly higher LI_Casp_-TIL compared to the conventional OSCC (Table 3). We also examined the correlation between TRAIL and TRAIL-R expression levels and apoptosis rate among tumor cells and tumor infiltrating lymphocytes (Table 3). There was no significant relationship between the expression levels of all four TRAIL-R in tumor cells and their apoptosis rate (Table 3). Moreover apoptosis rates of TIL in OSCC did not differ significantly between TRAIL- positive and -negative tumors (Table 3).

## Discussion

We and others have demonstrated that OSCC cells are susceptible to TRAIL-induced apoptosis [7,10,33]. A Phase 1 clinical study of TRAIL-R agonist antibody (HGS-ETR1) in patients with advanced solid malignancies including head and neck cancer has proven its safety and biological activity as measured by durable stable disease in these patients [20]. Our study, however, is the first to characterize and compare alterations in TRAIL and TRAIL-R in normal oral epithelia, OPM and OSCC. Our data document the following important findings: 1. Normal oral epithelia constitutively expressed TRAIL, but its expression was progressively lost in OPM and OSCC. 2. Reduction in DcR2 expression level was noted frequently in OPM and OSCC compared to respective patient-matched uninvolved oral mucosa. 3. Expression levels of DR4, DR5 and DcR1 receptors were not significantly altered in OPM, primary OSCC or metastatic OSCC compared to patient-matched normal oral mucosa. 4. Expression of proapoptotic TRAIL-receptors DR4 and DR5 in OSCC seemed to depend, at least in part, on whether or not these receptors were expressed in their parental oral epithelia. 5. High DR5 expression in primary OSCC correlated significantly with larger tumor size. 6. There was no significant association between TRAIL-R expression and OSSC histology grade, nodal status, or apoptosis rates of tumor cells and TIL.

### Loss of TRAIL expression is an early event during oral carcinogenesis

The functional role of TRAIL, which is constitutively expressed in most normal tissues, is poorly understood. Recently, however, TRAIL has been shown to trigger apoptosis in transformed or dysplastic epithelial cells, suggesting a therapeutic potential as a chemopreventive agent against malignant progression of premalignancies [19,34,35]. OPM is considered a progenitor of OSCC and represents an intermediate step in the progression from NOM to OSCC [30]. The likelihood for development of OSCC in OPM is generally proportional to its dysplasia grade [30]. Our data revealed that TRAIL, which is expressed in normal oral epithelia, was progressively lost in OPM with increasing dysplasia grade. This was further supported by our microarray data in which TRAIL mRNA levels were 8-fold higher in NOM cells compared to OPM cells. Finally, our immunoblotting data confirmed that TRAIL protein was detectable only in NOM cells not in OPM cells. Similar loss of TRAIL expression during the progression from normal epithelia to malignancy has been reported in skin, esophageal and colon cancers [36-38].

The loss of individual TRAIL-R expression in malignant tumors has been attributed to chromosomal gene mutation/deletion (DR4 and DR5) or promotor methylation (DcR1 and DcR2) events [4]. However, gene mutation or promotor methylation is not implicated for the malignancy-specific down-regulation of TRAIL expression. Recent experimental findings indicate that loss of TRAIL expression during malignant transformation is not mediated by genetic aberration but by dysregulation of signal transduction pathways common to various cancers. TNF-α increases the susceptibility of breast cancer cells to chemotherapy by up-regulating TRAIL expression by promoter activation [39]. Similarly, retinoids and interferons exert their anticancer activity in breast cancer cells by enhancing TRAIL expression in these cells [40]. Hyperactivation of the phosphatidylinositol 3-kinase (PI-3K)/Akt pathway has been implicated in suppressing TRAIL expression in colon cancer cells [41]. Treatment of colon cancer cells with the PI-3K inhibitor Wortmannin rescues TRAIL expression in these cells and induces enterocyte-like differentiation  [41]. Interestingly, hyperactivation of PI-3K/AKT pathway via dysregulated EGFR signaling is an important and early event in the pathogenesis of OSCC [42]. Hence, it is plausible that there is a cause-and-effect relationship between dysregulated prosurvival PI-3K/AKT signaling and loss of TRAIL expression in OPM and OSCC.

Interestingly, oral carcinogens such as tobacco smoke frequently inactivate the tumor suppressor gene *p53*, thus depriving its protective role against oral carcinogen-induced DNA damage [43]. TRAIL-induced apoptosis of transformed/dysplastic cells is independent of *p53 *status [34,44], hence, TRAIL may act as a substitute guardian against malignant transformation by eliminating transformed cells during the initial genesis OPM. Thus, TRAIL down-regulation may allow clonal expansion of transformed cells by protecting them from apoptosis, thereby increasing the risk of malignant progression.

TRAIL protein detection was mostly negative or only focally positive in isolated tumor cells in both primary and metastatic OSCC tumors, and our Western blot analysis clearly demonstrated the lack of TRAIL protein in primary and metastatic OSCC cell lines. Our microarray data also confirmed low levels of TRAIL mRNA in primary and metastatic OSCC cell lines compared to NOM. Our findings are not, however, in agreement with a previous study reporting the expression of TRAIL protein in primary OSCC tumor specimens and TRAIL mRNA expression in established OSCC cell lines [24,25]. That study used RT-PCR, which can presumably detect much lower levels of TRAIL mRNA compared to our microarray analysis. Moreover, that study did not compare the TRAIL mRNA levels of OSCC cell lines with NOM to determine significant differences in their respective expression levels. The microarray chip used in our study included three different probe sets for TRAIL which further increased the specificity and reliability of the data. Nevertheless, tumor heterogeneity and the differences in the specificity of the TRAIL antibody used in the two investigations may account for the discrepancies in the OSCC TRAIL expression data.

### Expression of DR4, DR5 and DcR1 receptors are not significantly altered during oral cancer progression

Relative expression levels of TRAIL death and decoy receptors are critical for exploiting therapeutic possibilities based on TRAIL/TRAIL-R interaction in OPM and OSCC [7,10,33,45]. Expression of these three receptors by OSCC seems to depend at least in part on whether or not they are expressed by the parental oral epithelium from which the tumors arose. Moreover, expression of all three receptors in the tumor cells were higher than in adjacent NOM, a feature also reported for melanomas and breast and colon carcinomas [46-48]. In contrast, DcR2 expression was significantly lower in OSCC compared to the adjacent NOM. Loss of DcR2 expression in malignant tumor cells has been attributed to aberrant promotor methylation [49].

DcR1, DR4 and DR5 are very frequently overexpressed in dysplastic oral mucosa, as well as primary and metastatic OSCC tumor specimens. All primary OSCC specimens examined by us expressed either DR4 or DR5 or both at high levels. Among the death-inducing receptors, DR4 was expressed more frequently in OSCC than DR5, as also observed by others in head and neck squamous cell carcinomas  [25]. Therefore, it would seem that the DR4-targeted humanized agonist antibody (HGS-ETR1) would be a better choice than the DR5-targeted antibody (HGS-ETR2) for OSCC clinical trials.

TRAIL death-inducing receptors DR4 and DR5 are considered tumor suppressors because their loss is expected to provide a survival advantage to the tumor cells. Conversely, overexpression of the anti-apoptotic decoy receptors DcR1 and DcR2 is expected to promote malignant progression. Therefore, we evaluated the association between the expression levels of these receptors and certain accepted prognostic indicators such as tumor size, nodal status and histologic tumor grade. Expression levels of these receptors failed to show any significant correlation with either nodal status or tumor grade, and there was no significant association between DR4, DcR1 and DcR2 expression levels and tumor size. However, DR5 expression correlated positively with tumor size. Such an association is in conflict with its proposed pro-apoptotic and tumor-suppressive function yet a similar observation is also made in breast cancers, in which high DR5 expression has correlated with nodal status, tumor size, and poor survival rate [48]. Similarly, high DR5 but not DR4 expression correlated with decreased survival in patients with non-small-cell lung cancers [50]. DR5 expression is induced by DNA damage and the induction is both wild-type *p53 *dependent and independent [51]. Activation of endogenous NF-kB factors by TNF-α also induces DR5 expression in tumor cells [52]. Thus, anti-apoptotic NF-kB activation may explain the association between increased DR5 expression and larger tumor size.

### TRAIL and TRAIL-R expression in OSCC does not influence tumor cell and tumor infiltrating lymphocyte apoptosis rates

Susceptibility of OSCC cells to TRAIL-mediated apoptosis is dependent on their relative expression levels of death-inducing receptors DR4 and DR5 versus decoy receptors DcR1 and DcR2. OSCC expressing high levels of DR4 and/or DR5 will be more susceptible to apoptosis than tumors expressing low levels of these receptors. Conversely, OSCC expressing high levels of decoy receptors often shows reduced tumor cell apoptosis rates. However, our data did not show any significant difference in the tumor cell apoptosis rates for primary OSCC with high and low levels of DR4 and/or DR5 expression, or for differential expression levels of decoy receptors DcR1 and DcR2.

TIL represent one of the manifestations of host immune response against malignancy [53], and several clinical studies have documented the prognostic significance of TIL in various malignancies, including OSCC [54,55]. Apoptosis of TIL in OSCC leads to the depletion of lymphocytes and negatively affects their prognosis [54]. This is further supported by our finding that TIL apoptosis rates in basaloid squamous cell carcinomas, a very aggressive variant of OSCC [56,57], were 5-fold higher than in conventional OSCC. However, TRAIL/TRAIL-R expression patterns or tumor cell apoptosis rates were not significantly different between basaloid squamous cell carcinoma and conventional OSCC. Depletion of TIL in malignant tumors is mediated by a counterattack against Fas-bearing lymphocytes by FasL-expressing tumor cells [58]. Therefore, the potential of tumor-cell-derived TRAIL cytotoxicity against TIL has been proposed by some studies [59]. However, our study did not find any significant difference in the TIL apoptosis rate between focal TRAIL-positive and TRAIL-negative OSCC.

## Conclusion

Our investigation has shown that TRAIL is constitutively expressed in normal oral mucosa but its expression is gradually lost in oral premalignant and malignant epithelia. OSCC more frequently expresses DcR1, followed by DR4 and DR5. Moreover, expression of DR4, DR5 and DcR1 are up-regulated in premalignant and malignant oral epithelia compared to normal oral epithelium. A partial loss of DcR2 expression is also noted in premalignant and malignant oral epithelia compared to normal oral epithelia, and DR5 expression is significantly associated with larger tumor size. Expression levels of TRAIL receptors show no significant correlation with nodal status and apoptosis rates of tumor cells and TIL.

## Abbreviations

TRAIL, tumor necrosis factor-related apoptosis-inducing ligand; NOM, normal oral mucosa; OPM, oral premalignancies, OSCC, oral squamous cell carcinoma; TIL, tumor-infiltrating lymphocytes; HRP, horseradish peroxidase, LI-Casp, caspase 3 labeling indices.

## Competing interests

The author(s) declare that they have no competing interests.

## Authors' contributions

NV and WZ planned and designed the studies, analyzed the data and drafted the manuscript. DB performed all immunohistochemical staining. JW performed cell culture, protein, RNA isolation and immunoblotting. JB and SM were involved in pathologic interpretation, sample collection and revising the manuscript. YL assisted in experimental designs and data interpretation methods. All authors read and approved the final manuscript.

## Pre-publication history

The pre-publication history for this paper can be accessed here:


